# Carotid arterial stiffness is associated with selective glomerular hypofiltration of middle-sized molecules in type 2 diabetes

**DOI:** 10.1177/14791641261493132

**Published:** 2026-09-28

**Authors:** Agne Laucyte-Cibulskiene, Elisabeth Kurien Thomas, Isak Lindberg, Peter M. Nilsson, Anders Christensson, Jan Nilsson, Michaela Kozakova, Carmela Morizzo, Giuseppe Penno, Monia Garofolo, Carlo Palombo

**Affiliations:** 1 Department of Clinical Sciences Malmö, 5193 Lund University, Malmö, Skåne County, Sweden; 2 Department of Nephrology, Skane University Hospital, Malmö, Skåne County, Sweden; 3 Faculty of Medicine, 5193 Lund University, Lund, Skåne County, Sweden; 4 Department of Clinical and Experimental Medicine, 9310 University of Pisa, Pisa, Italy; 5 Department of Surgical, Medical, Molecular Pathology and Critical Care Medicine, 9310 University of Pisa, Pisa, Italy; 6 University of Pisa and Pisa University Hospital, Cardiology Division, Pisa, Italy

**Keywords:** aortic stiffness, diabetes, local arterial stiffness, estimated glomerular filtration rate, selective glomerular hypofiltration syndromes

## Abstract

**Background:**

Type 2 diabetes (T2D) is a major driver of premature vascular aging. We investigated whether Selective Glomerular Hypofiltration Syndromes (SGHS), expressed as ratios of cystatin C– or β2-microglobulin–based estimated glomerular filtration rate to creatinine-based eGFR (eGFRcys/eGFRcr and eGFRb2M/eGFRcr), are associated with carotid, femoral, or aortic stiffness.

**Methods:**

We studied 144 individuals with T2D recruited from the University Diabetology Unit of Pisa, Italy. Local arterial stiffness was assessed at the carotid and femoral arteries using β stiffness index, Peterson’s elastic modulus (Ep), and local wave speed (WS). Systemic stiffness was evaluated by carotid–femoral pulse wave velocity (cfPWV) and augmentation index (AIx). Kidney function was assessed using eGFRcr, eGFRcys, eGFRb2M, mean eGFR, urinary albumin-to-creatinine ratio (UACR), and eGFR ratios.

**Results:**

A 0.1-unit decrease in eGFRcys/eGFRcr showed a borderline association with higher cfPWV (p=0.087), while age, diabetes duration, UACR, and central pulse pressure were stronger predictors. Lower eGFR ratios were associated with higher carotid WS in unadjusted analyses; after adjustment, eGFRcys/eGFRcr showed a nonsignificant trend (p=0.074). No associations were found with femoral stiffness indices.

**Conclusions:**

SGHS was inversely associated with carotid WS in T2D but not with aortic or femoral stiffness.

**Key Messages**

**What is currently known about this topic?**

The thickening of microvascular membranes in type 2 diabetes (T2D) including the kidneys reduces the permeability of middle-sized plasma molecules through glomerular basement membrane, thereby accelerating vascular remodeling.

**What is the key research question?**

Does the selectivity of the glomerular filtration barrier for cystatin C and β2-microglobulin reflects vascular stiffness, as measured by local arterial (carotid and femoral) and aortic stiffness in T2D?

**What is new?**

Size-selective glomerular filtration reflects more accurately the local arterial stiffness than aortic stiffness in T2D. The asynchronous progression of vascular ageing at different sites in the arterial tree in T2D suggests reduced protection of highly perfused organs, including the kidneys, potentially increasing susceptibility to end-organ damage.

**How might this study influence clinical practice?**

Findings suggest that evaluating kidney function by calculating the eGFRcys/eGFRcr or eGFRβ2m/eGFRcr ratios in T2D may improve the detection of vascular complications and thereby help identify individuals who need more aggressive cardiovascular disease prevention.

## 1. Introduction

The prevalence of type 2 diabetes (T2D) has been rapidly increasing over the past few decades.^1^ T2D and its related complications impose a significant morbidity and mortality risk but also an economic burden on the healthcare systems, leading to an overwhelming workload.^2^ Diabetic vascular diseases encompass a range of angiopathies affecting different levels of the vascular tree, including the aorta, renal arteries, peripheral arteries, coronaries, vertebrobasilar artery, and microvasculature in target organs, among others.^3,4^ The common underlying feature of arterial lesions is the thickening of the vascular basement membrane, which makes T2D a panvasculopathy.^4,5^

The bidirectional vascular–kidney interaction is well-known and may reflect this shared panvasculopathy, with asynchronous involvement of renal microvasculature and large arteries. Microvascular alterations are known to occur prior to the clinical diagnosis of T2D, however, substantial interindividual variability may exist in the onset and progression of changes within larger vascular beds. In the kidneys, vascular membrane is referred to as the glomerular basement membrane (GBM). The GBM, in conjunction with fenestrated glomerular endothelial cells and podocytes, forms the glomerular filtration barrier (GFB). The GFB acts as a charge- and size-selective gatekeeper, for filtration with a cut-off around 45 kDa in molecular mass. This ensures that medium-sized and large molecules remain in circulation while water, small-sized molecules, and solutes are filtered freely.^6^ The sieving coefficient decreases sharply at a molecular mass of 45 kDa in healthy kidneys. This means that eGFR for a small molecule like creatinine (∼113 Da) is similar to a medium-sized molecule like cystatin C (∼13,300 Da) or β2-microglobulin (∼11,800 Da) in most cases. However, in 8-10% of the adult population eGFRcys is 70% of eGFRcr despite normal kidney function. This hypofiltration for medium-sized molecules is designated Selective Glomerular Hypofiltration Syndromes (SGHS).^7^ SGHS encompasses at least two subtypes: Shrunken Pore Syndrome^8^ and Elongated Pore syndrome.^9^ The condition is mathematically expressed as the ratio between estimated cystatin C and creatinine-based filtration (eGFRcys/eGFRcr). The accumulation of evidence supports SGHS predictive value of pregnancy-related complications,^10^ frailty,^11^ incident heart failure,^12^ kidney disease,^13^ cardiovascular and all-cause mortality.^14,15^

However, these observational studies have not yet clarified the mechanisms through which cardiovascular risk is increased in individuals with SGHS. Is it due to functional changes in the vascular tree – such as endothelial dysfunction, oxidative stress, inflammation, and arterial stiffening – caused by the accumulation of atherosclerotic compounds^16^ and advanced glycation end products (AGEs),^17^ which then lead to subclinical atherosclerosis^18^? Could this hypothetical pathway be triggered by thickening of the vascular membrane – the GBM – a key histological feature observed in both SGHS and diabetic kidney disease (DKD)^19,20^? Against the background of this research gap, this study was conducted to investigate how the selectivity of the glomerular filtration barrier for cystatin C and β2-microglobulin reflects vascular stiffness, as measured by local arterial (carotid, femoral) and aortic stiffness in T2D (Figure 1). Early detection and intervention of vascular complications can significantly mitigate the burden of cardiovascular morbidity and mortality.

**Figure 1.**
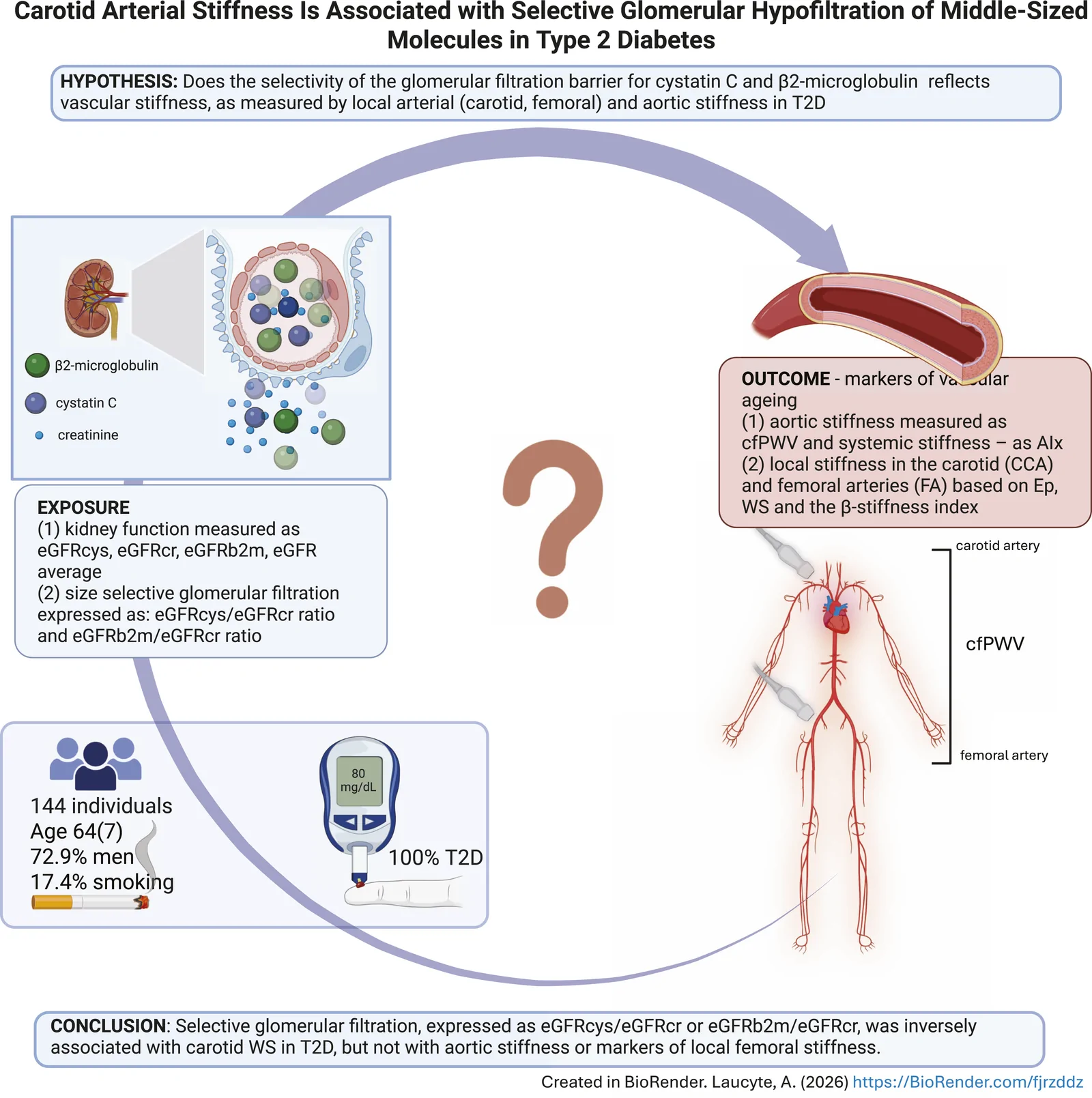
The Graphical Poster.

## 2. Methods

### 2.1. Study population

The study recruited 144 participants at the outpatient clinic of Pisa University Hospital Cisanello, Pisa, Italy, through a random selection process. The inclusion criteria were as follows: age above 18 years and a diagnosis of T2D. The exclusion criteria were severe systemic disease, clinically overt heart disease, systemic inflammatory diseases, and pregnancy. The sex distribution of the participants was 73% males and 27% females.

To minimize selection bias, participants were consecutively recruited from the outpatient clinic. To reduce measurement bias, only experienced staff performed measurements of local and systemic arterial stiffness according to standardized protocols. Potential confounders, including age and sex, were adjusted for in multivariable analyses. Despite these measures, residual confounding cannot be completely excluded.

No formal sample size calculation was performed prior to the study. The study size (n = 144) was determined by the number of eligible participants attending the outpatient clinic during the study period. This sample is comparable to previous studies of arterial stiffness and sufficient for multivariable regression analyses. We acknowledge that smaller effects may not be detected.

### 2.2. Demographic variables

Smoking status was self-reported. Hypertension was defined as a known diagnosis and blood pressure ≥ 140/90 mmHg. Medication charts were reviewed to identify antihypertensives, statins, oral antidiabetic medications, insulin, and antiplatelet agents. Weight (kg) and height (cm) were measured using calibrated scales and body mass index (BMI) calculated (kg/m^2^). Blood tests were conducted after a 12-hour fast, including fasting plasma glucose (FPG), HbA1c, creatinine, cystatin C, β2-microglobulin, and lipid profiles (triglycerides (TG), high density lipoprotein cholesterol (HDL-c), low density lipoprotein cholesterol (LDL-c)).

### 2.3. Local arterial stiffness

Local stiffness of the carotid and femoral arteries was measured using the Hitachi-Aloka Prosound Alpha10 (Japan) ultrasound system, a validated device for non-invasive vascular assessment. Participants were scanned after a minimum of 10 minutes of rest in a supine position to ensure optimal relaxation. The right common carotid artery (CCA) was imaged using a high-frequency ultrasound transducer, with the left CCA used as a substitute if necessary (less than 10% of cases). The diameter waveform of the CCA was measured by tracking the movement of the anterior and posterior walls of the artery, with the measurements taken approximately 1 cm proximal to the carotid sinus. The measurements were calibrated using simultaneously recorded blood pressure readings from the brachial artery, as previously described.^19^ The ultrasound system quantified, and local wave speed (WS), and elasticity via Peterson’s elastic modulus (Ep) and β stiffness index, in accordance with established methodology.^21^

### 2.4. Systemic aortic stiffness

Systemic stiffness of the aorta was assessed using the feet-to-feet method, which involves measuring carotid-femoral pulse wave velocity (cfPWV) using Complior Analyze Software. This method involves simultaneous recording of pulse waves at the carotid artery and femoral artery sites, followed by processing of the signals to extract the time delay between the two sites. The cfPWV was then calculated by dividing the distance between the carotid and femoral pulse peak sites, typically measured using a tape, by the time delay. The augmentation index (AIx) calculated from carotid pulse wave analysis was used as a marker of systemic stiffness.

### 2.5. Kidney function

Kidney function was assessed using estimated glomerular filtration rates (eGFR) based on creatinine (eGFRcr), cystatin C (eGFRcys), and β2-microglobulin (eGFRb2M). The molecular masses of the markers are as follows: creatinine (∼113 Da), cystatin C (∼13,300 Da), and β2-microglobulin (∼11,800 Da). The eGFR equations employed in this study were the Lund-Malmö revised (LMrev) formula for creatinine, the Caucasian and Asian Pediatric Adult subjects (CAPA) for cystatin C, and the method proposed by Inker et al. for β2-microglobulin.^22–24^ The cystatin C and creatinine equations used in this study have been recognized as valid for evaluating kidney function by the Kidney Disease Improving Global Outcomes (KDIGO) guidelines ^25^**.** SGHS was calculated both as the ratio of eGFRcys/eGFRcr and eGFRb2M/eGFRcr. The average eGFR (eGFR average) was calculated as the mean of eGFRcys and eGFRcr. Urine albumin-to-creatinine (UACR) ratio in the morning spot urine was used to determine albumin excretion.

### 2.6. Statistical analysis

This cross-sectional study analyzed continuous data using normal distribution curves, except for albuminuria, which was transformed using a 10-logarithmic scale. Continuous data are presented as means (standard deviation) and categorical data as percentages (number). No missing data were observed in the study Pearson correlation and linear regression were used for association analysis between outcome and exposure variables. Due to the small sample size, logistic regression was not employed.

Univariate analysis was performed for systemic and local stiffness parameters, kidney function markers, and covariates (lipid status, hypertension, FPG, HbA_1c_, antiplatelets, oral antidiabetics, insulin, etc.). Variables significantly related to the outcome at p <0.1 were included in multivariable linear regression analysis, following the rule of ten (dependent variable adjusted for up to 14 variables). BMI was not significantly related to exposure variables or outcomes and was excluded from multiple models. Based on univariate linear regression and previous knowledge, we selected age, sex, smoking, T2D duration, HbA1c, FPG, TG, renin-angiotensin-aldosterone (RAAS) inhibiting medications, oral antidiabetic medications, insulin, antiplatelets, heart rate (HR), central pulse pressure (cPP), and albumin excretion as mandatory variables. Variation inflation factors were used to avoid multicollinearity, with levels under 2 accepted.

In our cohort cystatin C and β2-microglobulin concentrations could not be used in models together with eGFR due to high intercolinearity.

Due to small sample size only six individuals had eGFRcys/eGFRcr <0.7, and 16 individuals had values <0.8. Therefore, we used the cut-off value of 0.9 for group comparison.

Statistical analysis was performed using SPSS 29.0, with a p-value < 0.05 considered statistically significant.

## 3. Outcomes

Multiple linear regressions were performed in several models to examine the relationships between the exposures and multiple outcomes. Specifically, the outcomes included: (1) aortic stiffness measured as cfPWV and systemic stiffness – as AIx; and (2) local stiffness in the carotid (CCA) and femoral arteries (FA) based on Ep, WS and the β-stiffness index.

## 4. Exposures

In this study, the following kidney function markers were used as exposures: eGFRcys, eGFRcr, eGFRb2m, eGFR average, eGFRcys/eGFRcr ratio, and eGFRb2m/eGFRcr ratio.

## 5. Results

### 5.1. Participants

The cohort consisted of 144 T2D individuals (73% men). The average age was 64 years (SD ± 8). Notable sex differences included higher diastolic and central systolic blood pressure, LDL-c, and ln(UACR) in men (P < 0.05). Forty-seven individuals had eGFRcys/eGFRcr < 0.9, characterized by higher smoking and dyslipidemia prevalence, increased use of diuretics and insulin, and decreased use of oral antidiabetics. Despite similar diabetes duration and glycemic status, they had lower eGFRcys, eGFR average, eGFRcys/eGFRcr and eGFRb2M/eGFRcr. Vascular parameters were similar (Table 1).

**Table 1.** Descriptives of the study population.

Variable	Total population (n=144)	eGFRcys/eGFRcr<0.9 (n=47)	eGRcys/eGFRcr ≥0.9 (n=97)	p-value
***Demographic variables***				
Age, years	64(7)	64(7)	64(7)	0.909
Sex, males	72.9(105)	74.5(35)	72.2(70)	0.771
Body mass index, kg/m^2^	29.3(4.8)	30.1(5.9)	28.9(4.10)	0.143
Smoking, yes	17.4(25)	29.8(14)	12.4(12)	0.038
T2D duration, years	14.0(8.9)	15.6(10)	13.2(8.2)	0.122
Hypertension, yes	99.3(143)	83.0(39)	75.3(73)	0.296
Dyslipidemia, yes	66.0(95)	78.7(37)	59.8(58)	0.025
***Medications***				
ACEi	32.6(47)	38.3(18)	29.9(29)	0.313
ARB	31.9(46)	31.9(15)	32.0(31)	0.996
BB	23.6(34)	27.7(13)	21.6(21)	0.426
CCB	29.2(42)	36.2(17)	25.8(25)	0.198
Alpha-blockers	4.2(6)	4.3(2)	4.1(4)	0.970
Diuretics	27.1(39)	40.4(19)	20.6(20)	0.012
Statins	66.0(95)	76.6(36)	60.8(59)	0.061
Oral antidiabetics	79.2(114)	66.0(31)	85.6(83)	0.007
Insulin	30.5 (44)	44.7(21)	23.7(23)	0.010
Antiplatelet	33.3 (48)	34.0(16)	33.0(32)	0.900
***Biochemistry***				
HbA1c, %	7.0(1.1)	7.0(1.3)	7.0(0.9)	0.779
FPG, mg/dL	140(52)	138(58)	141(50)	0.747
HDL-c, mg/dL	49(14)	47(12)	49(16)	0.414
LDL-c, mg/dL	84(27)	80(26)	86(28)	0.200
Triglycerides, mg/dL	173(87)	167(86)	176(87)	0.578
***Local and systemic stiffness***				
CCA Aix, %	18.9(11.0)	20.3(22.8)	19.5(16.5)	0.826
CCA β stiffness index, units	10.7(4.3)	10.4(4.7)	10.8(4.1)	0.589
CCA Ep, kPa	146.6 (61.2)	142(69)	149(58)	0.490
CCA WS, cm/s	7.2(1.4)	7.0(1.5)	7.3(1.5)	0.356
FA β stiffness index, units	9.5(0.6)	10.2(8.9)	9.2(5.6)	0.434
FA Ep, kPa	132.9 (96.0)	141(131)	129(83)	0.520
FA WS, cm/s	6.6(0.2)	6.7(2.5)	6.6(2.0)	0.765
cfPWV, m/s	12.0(2.7)	12.3(3.2)	11.8(2.4)	0.376
cPP, mmHg	41(13)	39(13)	42(13)	0.240
HR, bpm	69(11)	70(12)	68(11)	0.429
***Kidney function***				
eGFRcys, mL/min/1.73m^2^	60.2(24.2)	48.5(18.0)	65.8(25.0)	<0.001
eGFRcr, mL/min/1.73m^2^	59.8(21.4)	59.7(20.0)	59.8(22.2)	0.976
eGFRb2M, mL/min/1.73m^2^	65.4(21.7)	58.0(17.7)	69.0(22.6)	0.004
eGFR average, mL/min/1.73m^2^	60.0 (22.1)	54.1 (18.8)	62.8 (23)	0.027
eGFRcys/eGFRcr ratio	1.01(0.20)	0.80(0.09)	1.11(0.17)	<0.001
eGFRb2M/eGFRcr ratio	1.14(0.24)	1.00(0.15)	1.20(0.25)	<0.001
Ln(UACR)	-0.9(2.0)	-0.4(2.2)	-1.1(1.9)	0.075

Table 1 presents a comparison of study variables between the group with eGFRcys/eGFRcr < 0.9 (n = 47) and the group with eGFRcys/eGFRcr ≥ 0.9 (n = 97). Continuous variables are presented as mean (SD), and categorical variables as percentages (number of cases). P-values were calculated using a two-tailed unpaired Student’s t-test for continuous variables and Fisher’s exact test for categorical variables.

Abbreviations: HbA1c – glycolyzed haemoglobin, FPG – fasting plasma glucose, HDL-c – high-density cholesterol, LDL-c – low-density cholesterol, CRP – C-reactive protein, CCA – common carotid artery; FA – femoral artery; Ep – Peterson’s elastic modulus, AIx – augmentation index, WS – local wave speed, cfPWV- carotid-femoral pulse wave velocity, cPP – central pulse pressure; HR – heart rate; eGFRcys – cystatin C based estimated glomerular filtration rate, eGFRcr – creatinine-based eGFR, eGFRb2M – β2-microglobulin based eGFR, eGFR average – mean of the sum of eGFRcys and eGFRcr, ln(UACR) – natural logarithm of urin albumin-to-creatinine ratio, ACEi – angiotensin-converting enzyme inhibitor, ARB – angiotensin II receptor blocker, BB – beta blockers, CCB – calcium channel blockers.

### 5.2. Systemic stiffness as an outcome

#### 5.2.1. Carotid-femoral pulse wave velocity

Unadjusted linear regression revealed that a 1 SD decrease in eGFRcys and eGFRb2M was significantly associated with increased cfPWV (Table 2). Average eGFR showed a similar trend, although with borderline significance. After adjusting for age, sex, smoking, T2D duration, and ln(UACR), previously observed relationships disappeared. Instead, we found that older age (p < 0.001), longer T2D duration (p < 0.001), and higher ln(UACR) (p < 0.05) were significantly associated with higher cfPWV in all models (Supplemental Table S1, Model 2 and 3).

**Table 2.** Summary of significant and borderline significant results from multiple linear regression analysis.

Kidney Marker	Model	cfPWV (β [95% CI], p)	AIx (β [95% CI], p)	Local CCA WS (β [95% CI], p)	Local FA WS (β [95% CI], p)
eGFRcys per SD increase	Unadjusted	-0.46 [-0.90, -0.01], p=0.046	n.s.	n.s.	-0.34 [-0.70, 0.03], p=0.073
​	Model 1	n.s.	n.s.	n.s.	-0.41 [-0.80, -0.02], p=0.038
​	Model 2	n.s.	n.s.	n.s.	n.s.
​	Model 3	n.s.	n.s.	n.s.	n.s.
eGFRcr per SD increase	Unadjusted	n.s.	n.s.	-0.22 [-0.44, 0.004], p=0.054	-0.47 [-0.82, -0.11], p=0.011
​	Model 1	n.s.	n.s.	n.s.	-0.58 [-0.96, -0.19], p=0.004
​	Model 2	n.s.	n.s.	n.s.	-0.48 [-0.90, -0.06], p=0.024
​	Model 3	n.s.	n.s.	n.s.	-0.53 [-0.97, -0.08], p=0.020
eGFRb2M per SD increase	Unadjusted	-0.50 [-0.95, -0.05], p=0.029	n.s.	n.s.	-0.38 [-0.74, -0.01], p=0.044
​	Model 1	n.s.	n.s.	n.s.	-0.45 [-0.84, -0.07], p=0.021
​	Model 2	n.s.	n.s.	n.s.	n.s.
​	Model 3	n.s.	n.s.	n.s.	n.s.
eGFRav per SD increase	Unadjusted	-0.41 [-0.86, 0.03], p=0.069	n.s.	n.s.	-0.41 [-0.77, -0.05], p=0.027
​	Model 1	n.s.	n.s.	n.s.	-0.51 [-0.90, -0.12], p=0.011
​	Model 2	n.s.	n.s.	n.s.	-0.40 [-0.83, 0.03], p=0.067
​	Model 3	n.s.	n.s.	n.s.	n.s.
eGFRcys/eGFRcr ratio	Unadjusted	-2.15 [-4.32, 0.02], p=0.052	-12.9 [-24.9, -0.9], p=0.035	0.99 [-0.08, 2.07], p=0.070	n.s.
​	Model 1	-2.4 [-4.4, -0.4], p=0.019	-13.9 [-25.8, -1.86], p=0.024	0.90 [-0.15, 1.95], p=0.093	n.s.
​	Model 2	n.s.	n.s.	1.25 [0.10, 2.41], p=0.033	n.s.
​	Model 3	-1.75 [-3.76, 0.26], p=0.087	n.s.	1.07 [-0.10, 2.24], p=0.074	n.s.
eGFRb2M/eGFRcr ratio	Unadjusted	-1.67 [-3.54, 0.20], p=0.079	n.s.	n.s.	n.s.
​	Model 1	-2.5 [-4.2, -0.7], p=0.005	n.s.	n.s.	n.s.
​	Model 2	n.s.	n.s.	n.s.	n.s.
​	Model 3	n.s.	n.s.	n.s.	n.s.

Table 2 presents Linear regression models assessing associations between the exposure variable and study outcomes in the overall study population (n = 144). Only models with a significance level of P < 0.1 are shown. P values were obtained from univariate and multivariate linear regression models. Detailed results for all significant multivariable linear regression models are provided in Supplementary Table S1. “n.s.” indicates non-significant models.

Model 1: exposure variable adjusted for age, sex

Model 2: Model 1 + additionally adjusted for smoking, T2D duration, ln(UACR)

Model 3: Model 2 + additionally adjusted for HbA1c, FPG, TG, use of renin-angiotensin-aldosterone affecting medications, oral antidiabetic medications, insulin, antiplatelets, HR, cPP

Abbreviations: HbA1c – glycolyzed haemoglobin, CCA – common carotid artery; FA – femoral artery; AIx – augmentation index, WS – local wave speed, cfPWV- carotid-femoral pulse wave velocity, eGFRcys – cystatin C based estimated glomerular filtration rate, eGFRcr – creatinine-based eGFR, eGFRb2M – β2-microglobulin based eGFR, eGFR average – mean of eGFRcys and eGFRcr, SD – standard deviation.

Model 3 showed a borderline association between cfPWV and eGFRcys/eGFRcr (p = 0.087), with a 0.1 unit decrease in eGFRcys/eGFRcr corresponding to a predicted 0.18 m/s increase in cfPWV (Table 2). Factors attenuating this relationship included older age (p < 0.001), higher HR (p < 0.001), and higher cPP (p < 0.001). Other study exposures did not show any relationship with cfPWV in adjusted analysis.

#### 5.2.2. Carotid augmentation index

Carotid arterial AIx was significantly influenced by lower eGFRcys/eGFRcr in unadjusted analysis (p = 0.035) and in Model 1 (p=0.024) (Table 2). A 0.1 decrease in eGFRcys/eGFRcr was associated with a 12.9% increase in carotid Aix adjusted for age and sex. However, Models 2 and 3 were not statistically significant. Other kidney markers were not related to Aix (Table 2).

### 5.3. Local arterial stiffness as an outcome

#### 5.3.1. Local wave speed

Unadjusted linear regression analysis revealed that lower eGFRcr, lower eGFRcys/eGFRcr, and lower eGFRb2M/eGFRcr were associated with higher carotid WS (Table 2). In Model 2, a 0.1-unit decrease in eGFRcys/eGFRcr predicted a 0.13 cm/s decrease in carotid WS. Other significant predictors of carotid WS were higher age (β = 0.05, p = 0.006) and higher log-transformed urinary albumin-to-creatinine ratio (ln UACR) (β = 0.21, p < 0.001) (Supplemental Table S1).

In Model 3, eGFRcys/eGFRcr showed a trend towards a positive relationship with carotid WS (p = 0.074), but older age (β = 0.05, p = 0.005), higher heart rate (HR) (β = 0.04, p < 0.001), and higher central pulse pressure (cPP) (β = 0.02, p = 0.032) were the significant factors related to higher WS (Supplemental Table S1).

Meanwhile, lower eGFRcr, lower eGFRb2M, and lower average eGFR were associated with higher femoral WS, while eGFRcys showed a trend towards a negative relationship (Table 2). Notably, a 1 SD decrease in eGFRcr was associated with a 0.53 cm/s increase in femoral WS (Table 2), which was not influenced by other factors included in Model 3 (Supplemental Table S1).

#### 5.3.2. Beta stiffness index and Peterson’s elastic modulus

Carotid beta stiffness index was not significantly influenced by the exposure variables in adjusted models, as age (β = 0.18, p < 0.001) and treatment with antiplatelets (β = 1.63, p = 0.030) were stronger predictors. Quite similar results observed with carotid Ep where age (β = 2.33, p < 0.001), and cPP (β = 1.56, p < 0.001) were found to be significant predictors. Additionally, none of the study exposures were associated with femoral beta stiffness index or femoral Ep after multiple adjustments. Results of univariate models are presented in Supplementary Table S2.

## 6. Discussion

Our analysis could not reveal the association between SGHS, expressed as eGFRcys/eGFRcr and eGFRb2M/eGFRcr ratios, and aortic stiffness, as measured by cfPWV. Age, longer T2D duration, higher urinary albumin excretion, and higher cPP were stronger predictors of aortic stiffness than SGHS. When analyzed separetaly, lower eGFRcys or eGFRb2M – indicating worse kidney function – was associated with higher cfPWV. However, this relationship disappeared when accounting for the influence of other factors, as previously indicated. Lower eGFRcys/eGFRcr ratio was related to systemic stiffness, expressed as higher AIx, adjusted for age and sex. A compelling finding was that lower eGFRcys/eGFRcr was significantly associated with lower carotid WS. This association was not influenced by factors such as age, sex, T2D duration, smoking, urinary albumin excretion, insulin treatment, or cPP. Furthermore, SGHS was not related to parameters of local femoral stiffness.

To the best of our knowledge, this is the second study to investigate the relationship between SGHS and vascular parameters in patients with T2D. Khang et al. analyzed carotid intima-media thickness (CIMT) and brachial-ankle pulse wave velocity (baPWV) in a cohort of 860 T2D patients. Their study found that a ratio of eGFRcys/eGFRcr below 0.9 was associated with higher baPWV and a higher prevalence of carotid intima media thickness (CIMT) ≥1.5 mm in either the left or right common carotid artery.^25^ Notably, the participants in their study were younger (mean age 55.2 ± 9.3 years), with a higher proportion of females, shorter duration of T2D, lower prevalence of smoking and hypertension, and greater variation in kidney function. However, they had higher hemoglobin A1C levels. Furthermore, only 81 participants in their study (9.4%) were classified as having SGHS, whereas in our cohort, 47 patients (32.6%) had eGFRcys/eGFRcr ratio below 0.9. Unfortunately, due to the high variability across cohorts and the use of different vascular parameters, the results are not directly comparable. Interestingly, Khang et al. found that SGHS could also predict the calculated 10-year atherosclerotic cardiovascular disease (ASCVD) risk.^25^ In general population-based cohort with a low prevalence diabetes, the relationship between the eGFRcys/eGFRcr ratio and aortic stiffness was not reported.^26^ In a sample of diabetes-free individuals, SGHS was related to subclinical atherosclerosis. These results demonstrated sex-specific differences, with the eGFRcys/eGFRcr ratio in women being associated with accelerated intima-media thickening of the carotid bulb, but not the common carotid artery, over 17 years.^18^ No other studies have explored the association between SGHS and vascular ageing. Vascular aging is a dynamic and longitudinal process, necessitating prospective studies with multiple measurements of markers reflecting SGHS to fully capture the changing relationship over time.

A striking finding is that lower eGFRcys/eGFRcr ratio significantly influenced local carotid stiffness, as represented by lower carotid WS. No interaction was found between these factors and variables such as age, sex, T2D duration, smoking, UACR, insulin treatment, or cPP, that could explain this unexpected finding. This result does not align with the proposed hypothesis that an increase in vascular aging, accompanied by an increase in WS, should be correlated with a decrease in selective glomerular hypofiltration.^27^ This finding can be interpreted in the context of previous studies that have observed a higher discrepancy between aortic stiffness and stiffness in first-generation arteries (e.g., carotid artery) in individuals with T2D compared to the control group, suggesting a higher risk of pulsatile damage to highly perfused organs such as the kidney, brain, heart, liver, and lungs.^28–30^ The accelerated stiffening of the proximal aorta (the most elastic segment) compared to distal segments and aortic branches in T2D, as measured by higher WS in our study, may imply a greater transmission of pulsatile flow and power, and reduced reflection in the distal carotid circulation.^29,30^ We could also observe older age, higher albumin excretion, alongside with lower eGFRcys/eGFRcr ratios only confirming that in this cohort lower WS could reflect rather a pathological condition. Furthermore, the femoral artery (a muscular artery) also responds to aortic stiffening by decreasing both reverse and diastolic forward flows, leading to visceral diastolic hypoperfusion, including the kidneys. This may explain the relationship between lower eGFR and higher WS.^31^ Nevertheless, the discrepancy between our findings and previous studies may also partly reflect the relatively modest sample size of our cohort, which may have limited our ability to fully capture the heterogeneity of vascular remodeling in T2D. Therefore, larger studies are needed to determine whether the observed association between lower eGFRcys/eGFRcr ratio and lower carotid WS represents a true biological phenomenon or variability related to cohort characteristics.

In addition, it is also possible that these findings are influenced by antidiabetic and antihypertensive treatment. Previous studies have found that incretin-based agents (DPP-4 inhibitors and GLP-1 receptor agonists) improved PWV, flow-mediated dilation, and perfused boundary region when HbA_1c_ levels were under 7%.^32^ Additionally, the protective effects of SGLT2 inhibitors on vascular and kidney function may also distort the observed relationship between SGHS and local WS. Other factors that may influence the relationship between eGFRcys/eGFRcr and WS include treatment with diuretics, which can increase blood viscosity and subsequently lead to lower WS,^33,34^ and a positive wave reflection coefficient together with tethering of the arteries which may also contribute to an underestimation of wave speed.^35,36^

Smoking is another important factor that may have influenced our results. Individuals with eGFRcys/eGFRcr ratios below 0.9 reported a significantly higher prevalence of current smoking. Previous analysis of 2,770 individuals found that active smoking was associated with higher eGFRcr levels but lower eGFRcys levels.^37^ Furthermore, smoking has been shown to worsen endothelial function and increase the risk of microvascular complications T2D by 2.77 times.^36^ Smoking-related nodular sclerosis, a histological finding in kidney biopsies, may mimic diabetes nephropathy since they share similar pathological findings, such as the formation of glomerular mesangial nodules, thickening of tubular basement membranes and GBM, and exudative lesions (arteriolar hyalinosis, fibrin cap, capsular drop).^38^ Smoking could be additional trigger behind GBM thickening in DKD which is also a histological hallmark of SGHS.^20^

An additional consideration is that tubular dysfunction, a well-recognized component of diabetic kidney disease, may influence the filtration markers used to define SGHS. Creatinine is eliminated through both glomerular filtration and tubular secretion, and alterations in tubular secretion may affect plasma creatinine concentrations independently of glomerular filtration rate. β2-microglobulin is a middle-sized molecule that readily passes the glomerular filtration barrier and is subsequently taken up and degraded by proximal tubular cells.^39,40^ As a result, elevated urinary β2-microglobulin levels are commonly regarded as evidence of impaired proximal tubular reabsorptive capacity. Previous studies have reported increased urinary β2-microglobulin excretion in individuals with T2D, indicating that tubular injury may contribute to altered β2-microglobulin handling.^40^ Consequently, abnormalities in proximal tubular function, together with changes in creatinine secretion, could influence eGFRb2M/eGFRcr and potentially eGFRcys/eGFRcr ratios independently of glomerular filtration, suggesting that these measures may capture aspects of tubulointerstitial injury in addition to selective glomerular hypofiltration.^39,40^

## 7. Strengths and limitations

A limitation of this study is the unequal sex distribution, with 73% males and 27% females, which may lead to selection bias and non-representative sampling. However, this imbalance may be justified by the higher prevalence of diabetes among Italian men aged 45-74.^41^ Methodological limitations also exist, including the potential for carotid artery stiffness to underestimate total aortic stiffness, particularly in patients with diabetes and hypertension.^28^ Our study population may therefore yield inconsistent results. The cross-sectional design of the study is another limitation, as it lacks prospective data and makes it difficult to determine the direction of the relationship between the two factors. Additionally, the ethnically non-diverse sample consisting only of Italian-born participants may limit the generalizability of the results. Finally, although fenofibrate treatment may increase serum creatinine concentrations and thereby influence creatinine-based eGFR estimates, only three participants (2.1%) in our cohort were receiving fibrate therapy, making it unlikely that this drug materially affected our results.

However, using a sample of patients with diabetes is a strength of this study, as it allows for the exploration of factors that have been previously understudied in this patient group. In contrast, using a healthy elderly sample would introduce uncontrolled variables, such as comorbidities, which are avoided in this study. Another strength is the use of well-established methods for measuring arterial stiffness and eGFR, which minimizes the risk of information bias. The gold-standard method for estimating arterial stiffness is cfPWV, and the equations for eGFR are approved by the KDIGO international guidelines.^42^

## 8. Conclusion

SGHS, expressed as eGFRcys/eGFRcr or eGFRb2m/eGFRcr, was associated with carotid WS in T2D, and particularly with systemic stiffness, but not with aortic stiffness or markers of local femoral stiffness. The influence on aortic stiffness was of borderline significance, likely due to the influence of other factors such as age, longer T2D duration, higher urinary albumin excretion, and higher cPP. The disparity between local arterial stiffness in the carotid and femoral arteries and aortic stiffness may be attributed to the heterogeneous elastic properties of these arteries and the asynchronous progression of vascular ageing at different sites within the arterial tree. This discrepancy may also suggest altered protection of highly perfused organs, potentially conferring increased susceptibility to end-organ damage.

## Supplemental material

Supplemental Material - Carotid arterial stiffness is associated with selective glomerular hypofiltration of middle-sized molecules in type 2 diabetes Supplemental Material for Carotid arterial stiffness is associated with selective glomerular hypofiltration of middle-sized molecules in type 2 diabetes by Agne Laucyte-Cibulskiene, Elisabeth Kurien Thomas, Isak Lindberg, Peter M. Nilsson, Anders Christensson, Jan Nilsson, Michaela Kozakova, Carmela Morizzo, Giuseppe Penno, Monia Garofolo, Carlo Palombo in Diabetes & Vascular Disease Research
