# Supplementary material for: Carotid arterial stiffness is associated with selective glomerular hypofiltration of middle-sized molecules in type 2 diabetes
Source: Diab Vasc Dis Res. 2026 Sep 28;23(5):14791641261493132. doi: 10.1177/14791641261493132 (PMC13735471; doi:10.1177/14791641261493132)
Supplement: Supplementary file 1 [file sj-pdf-1-dvr-10.1177_14791641261493132.pdf]

Supplemental Table S1. Nested multivariable regression models (Models 2 and 3) showing significant and borderline significant associations.

**MODEL 2**				95% Confidence interval	
**Outcome - cfPWV**	Beta	SE	p-value	Lower	Higher
Age	0.123	0.032	<.001	0.06	0.187
Sex, male	0.792	0.464	0.09	-0.126	1.711
Smoking, yes	-0.208	0.295	0.481	-0.792	0.375
T2D duration	0.091	0.024	<.001	0.044	0.138
Ln(UACR)	0.238	0.113	0.037	0.014	0.462
1SD increase in eGFRcys	0.11	0.232	0.637	-0.349	0.569
Age	0.126	0.033	<.001	0.061	0.192
Sex, male	0.796	0.463	0.088	-0.12	1.712
Smoking, yes	-0.221	0.294	0.455	-0.803	0.362
T2D duration	0.089	0.024	<.001	0.042	0.136
Ln(UACR)	0.241	0.112	0.032	0.02	0.462
1SD increase in eGFRcr	0.146	0.231	0.53	-0.312	0.603
Age	0.118	0.032	<.001	0.055	0.181
Sex, male	0.829	0.472	0.082	-0.105	1.764
Smoking, yes	-0.203	0.297	0.496	-0.79	0.385
T2D duration	0.091	0.024	<.001	0.044	0.138
Ln(UACR)	0.232	0.116	0.048	0.002	0.463
1SD increase in eGFRb2M	0.06	0.235	0.798	-0.404	0.524
Age	0.125	0.033	<.001	0.06	0.19
Sex, male	0.793	0.464	0.09	-0.125	1.71
Smoking, yes	-0.213	0.294	0.471	-0.796	0.37
T2D duration	0.09	0.024	<.001	0.043	0.137
Ln(UACR)	0.241	0.113	0.034	0.018	0.465
1SD increase in eGFR average	0.132	0.233	0.573	-0.329	0.592
Age	0.12	0.03	<.001	0.061	0.179
Sex, male	0.804	0.462	0.084	-0.11	1.718
Smoking, yes	-0.261	0.298	0.383	-0.85	0.328
T2D duration	0.086	0.024	<.001	0.038	0.134
Ln(UACR)	0.199	0.109	0.069	-0.016	0.415
eGFRcys/eGFRcr	-1.012	1.085	0.353	-3.158	1.134
Age	0.126	0.031	<.001	0.066	0.187
Sex, male	0.866	0.465	0.065	-0.055	1.786
Smoking, yes	-0.215	0.294	0.467	-0.797	0.368
T2D duration	0.081	0.025	0.001	0.032	0.129
Ln(UACR)	0.211	0.106	0.049	0.001	0.421

eGFRb2M/eGFRcr	-1.381	0.921	0.136	-3.203	0.441
**Outcome – CCA WS**					
Age	0.046	0.016	0.006	0.014	0.078
Sex, male	0.234	0.248	0.347	-0.257	0.725
Smoking, yes	-0.093	0.162	0.566	-0.415	0.228
T2D duration	-0.009	0.013	0.51	-0.035	0.017
Ln(UACR)	0.206	0.059	<.001	0.089	0.323
eGFRcys/eGFRcr	1.254	0.583	0.033	0.101	2.407
**Outcome – FA WS**					
Age	-0.034	0.03	0.259	-0.095	0.026
Sex, male	0.762	0.416	0.069	-0.061	1.584
Smoking, yes	-0.035	0.269	0.898	-0.568	0.498
T2D duration	0.01	0.022	0.649	-0.034	0.054
Ln(UACR)	0.135	0.102	0.187	-0.066	0.336
1SD increase in eGFRcr	-0.483	0.212	0.024	-0.903	-0.063
Age	-0.027	0.03	0.367	-0.087	0.032
Sex, male	0.779	0.419	0.065	-0.05	1.608
Smoking, yes	-0.058	0.271	0.832	-0.594	0.479
T2D duration	0.006	0.022	0.783	-0.038	0.05
Ln(UACR)	0.139	0.103	0.183	-0.066	0.343
1SD increase in eGFR average	-0.399	0.216	0.067	-0.826	0.028

**MODEL 3**				95% Confidence interval	
**Outcome - cfPWV**	Beta	SE	p-value	Lower	Higher
Age	0.133	0.031	<.001	0.072	0.194
Sex, male	0.331	0.429	0.442	-0.519	1.181
Smoking, yes	-0.172	0.264	0.516	-0.695	0.351
T2D duration	0.072	0.025	0.004	0.023	0.121
HbA1c	-0.005	0.218	0.98	-0.437	0.427
FPG	-0.006	0.004	0.136	-0.015	0.002
TG	0.001	0.002	0.667	-0.004	0.006
Ln(UACR)	0.058	0.113	0.607	-0.165	0.281
RAAS inhibitors, yes	-0.658	0.424	0.124	-1.497	0.182
Oral antidiabetics, yes	0.434	0.536	0.42	-0.628	1.495
Insulin, yes	0.1	0.489	0.839	-0.868	1.067
Antiplatelet, yes	-0.219	0.409	0.594	-1.029	0.592
HR	0.077	0.019	<.001	0.04	0.114
cPP	0.092	0.016	<.001	0.06	0.124

eGFRcys/eGFRcr	-1.748	1.013	0.087	-3.755	0.259
**Outcome – CCA WS**					
Age	0.051	0.018	0.005	0.016	0.086
Sex, male	0.155	0.247	0.533	-0.335	0.644
Smoking, yes	-0.051	0.154	0.743	-0.356	0.255
T2D duration	-0.017	0.014	0.248	-0.045	0.012
HbA1c	0.093	0.128	0.472	-0.162	0.347
FPG	0	0.002	0.869	-0.005	0.004
TG	0.001	0.001	0.535	-0.002	0.004
Ln(UACR)	0.105	0.066	0.114	-0.025	0.236
RAAS inhibitors, yes	-0.129	0.247	0.603	-0.619	0.361
Oral antidiabetics, yes	0.336	0.308	0.278	-0.274	0.947
Insulin, yes	-0.05	0.282	0.86	-0.609	0.509
Antiplatelet, yes	0.311	0.234	0.186	-0.152	0.775
HR	0.038	0.011	<.001	0.017	0.059
cPP	0.02	0.009	0.032	0.002	0.039
eGFRcys/eGFRcr	1.069	0.592	0.074	-0.103	2.241
**Outcome – FA WS**					
Age	-0.041	0.034	0.225	-0.108	0.026
Sex, male	0.673	0.44	0.129	-0.198	1.544
Smoking, yes	0.055	0.273	0.84	-0.486	0.597
T2D duration	0.009	0.025	0.708	-0.039	0.058
HbA1c	0.097	0.239	0.685	-0.376	0.57
FPG	0	0.004	0.98	-0.008	0.009
TG	0.003	0.002	0.213	-0.002	0.008
Ln(UACR)	0.039	0.116	0.739	-0.191	0.269
RAAS inhibitors, yes	-0.248	0.442	0.576	-1.123	0.627
Oral antidiabetics, yes	0.894	0.565	0.116	-0.225	2.013
Insulin, yes	0.146	0.51	0.775	-0.863	1.156
Antiplatelet, yes	-0.199	0.422	0.637	-1.035	0.636
HR	0.026	0.019	0.166	-0.011	0.064
cPP	0.023	0.017	0.182	-0.011	0.056
1SD increase in eGFRcr	-0.531	0.226	0.02	-0.978	-0.084

Supplemental Table S1 presents significant and borderline significant nested linear regression Models 2 and 3 in 144 patients with type 2 diabetes. P values were obtained from multivariable linear regression models.

Abbreviations: HbA1c – glycolyzed haemoglobin, FPG – fasting plasma glucose, HDL-c – high-density cholesterol, LDL-c – low-density cholesterol, CRP – C-reactive protein, CCA – common carotid artery; FA – femoral artery; Ep – Peterson’s elastic modulus, AIx – augmentation index, WS – local wave speed,

cfPWV- carotid-femoral pulse wave velocity, cPP – central pulse pressure; HR – heart rate; eGFRcys – cystatin C based estimated glomerular filtration rate, eGFRcr – creatinine-based eGFR, eGFRb2M – β2-microglobulin based eGFR, eGFR average – mean of the sum of eGFRcys and eGFRcr, ln(UACR) – natural logarithm of urin albumin-to-creatinine ratio, ACEi – angiotensin-converting enzyme inhibitor, ARB – angiotensin II receptor blocker, BB – beta blockers, CCB – calcium channel blockers.

Supplemental Table S2. Univariate linear regression analyses showing significant and borderline significant associations with carotid and femoral β-stiffness index and Peterson's elastic modulus.

	**CCA beta stiffness**		**FA beta stiffness**		**CCA Ep**		**FA Ep**	
	Beta	p-value	Beta	p-value	Beta	p-value	Beta	p-value
eGFRcys	n.s.	n.s.	-0.05	0.039	n.s.	n.s.	-0.62	0.085
eGFRcr	-0.04	0.010	-0.07	0.007	-0.47	0.05	-0.95	0.017
eGFRcys/eGFRcr	n.s.	n.s.	n.s.	n.s.	n.s.	n.s.	n.s.	n.s.
eGFRb2M	n.s.	n.s.	-0.06	0.019	n.s.	n.s.	-0.8	0.045
eGFRb2M/eGFRcr	2.62	0.080	n.s.	n.s.	n.s.	n.s.	n.s.	n.s.
eGFRaverage	-0.03	0.042	-0.06	0.014	n.s.	n.s.	-0.82	0.036

Supplemental Table S2 presents beta coefficients and p-values from significant and borderline significant univariate linear regression analyses in 144 patients with type 2 diabetes. P values were obtained from univariate linear regression models.

Abbreviations: n.s. – non significant model, eGFRcys – cystatin C based estimated glomerular filtration rate, eGFRcr – creatinine-based eGFR, eGFRb2M – β2-microglobulin based eGFR, eGFR average – mean of the sum of eGFRcys and eGFRc
